# Turning the tables on cytomegalovirus: targeting viral Fc receptors by CARs containing mutated CH2–CH3 IgG spacer domains

**DOI:** 10.1186/s12967-018-1394-x

**Published:** 2018-02-08

**Authors:** Julia Proff, Charlotte U. Brey, Armin Ensser, Wolfgang Holter, Manfred Lehner

**Affiliations:** 1 Children’s Cancer Research Institute, Zimmermannplatz 10, 1090 Vienna, Austria; 2 Institute for Clinical and Molecular Virology, Universitätsklinikum Erlangen, Schlossgarten 4, 91054 Erlangen, Germany; 3 St. Anna Kinderspital, Department of Pediatrics, Medical University of Vienna, Kinderspitalgasse 6, 1090 Vienna, Austria

**Keywords:** Cytomegalovirus, Chimeric antigen receptor (CAR), Glycoprotein B (gB), Fc receptors, CH2–CH3 domain, IgG, Interferon gamma (IFN-γ), Tumor necrosis factor (TNF)

## Abstract

**Background:**

During infection with human cytomegalovirus (HCMV) several viral proteins occur on cell surfaces in high quantity. We thus pursue an HLA-independent approach for immunotherapy of HCMV using chimeric antigen receptors (CARs) and bispecific BiTE^®^ antibody constructs. In this context, HCMV-encoded proteins that mediate viral immune evasion and bind human IgG might represent particularly attractive target antigens. Unlike in observations of similar approaches for HIV and hepatitis B and C viruses, however, HCMV-infected cells develop a striking resistance to cytotoxic effector functions at later stages of the replication cycle. In our study we therefore wanted to test two hypotheses: (1) CAR T cells can efficiently inhibit HCMV replication independently from cytotoxic effector functions, and (2) HCMV can be targeted by CH2–CH3 IgG spacer domains that contain mutations previously reported to prevent exhaustion and to rescue CAR T cell function in vivo.

**Methods:**

Replication of GFP-encoding recombinant HCMV in fibroblasts in the presence and absence of supernatants from T cell co-cultures plus/minus cytokine neutralizing antibodies was analyzed by flow cytometry. CARs with wild type and mutated CH2–CH3 domains were expressed in human T cells by mRNA electroporation, and the function of the CARs was assessed by quantifying T cell cytokine secretion.

**Results:**

We confirm and extend previous evidence of antiviral cytokine effects and demonstrate that CAR T cells strongly block HCMV replication in fibroblasts mainly by combined secretion of IFN-γ and TNF. Furthermore, we show that fibroblasts infected with HCMV strains AD169 and Towne starting from day 3 have a high capacity for binding of human IgG1 and also strongly activate T cells expressing a CAR with CH2–CH3 domain. Importantly, we further show that mutations in the CH2–CH3 domain of IgG1 and IgG4, which were previously reported to rescue CAR T cell function by abrogating interaction with endogenous Fc receptors (FcRs), still enable recognition of FcRs encoded by HCMV.

**Conclusions:**

Our findings identify HCMV-encoded FcRs as an attractive additional target for HCMV immunotherapy by CARs and possibly bispecific antibodies. The use of specifically mutated IgG domains that bind to HCMV-FcRs without recognizing endogenous FcRs may supersede screening for novel binders directed against individual HCMV-FcRs.

**Electronic supplementary material:**

The online version of this article (10.1186/s12967-018-1394-x) contains supplementary material, which is available to authorized users.

## Background

The infection and reactivation of human cytomegalovirus (HCMV) continues to be a major cause of morbidity and mortality after hematopoietic stem cell transplantation (HSCT) and solid organ transplantation (SOT) [1–4]. Although effective drugs have been available for many years, and novel drugs have been introduced recently, there is still a need for more effective and less toxic approaches for treatment of HCMV [4–7]. HCMV is a highly immunogenic virus and is controlled in immunocompetent individuals by a durable immune response with a strong expansion of memory T cells [8]. This fact was exploited early on for the development of several strategies for ex vivo enrichment of HCMV-specific memory T cells, which have proven their efficacy in adoptive immunotherapy after HSCT and even SOT [9–12].

We pursue an HLA-independent immunotherapeutic approach of targeting HCMV proteins by chimeric antigen receptors (CARs) and bispecific antibodies [13] (and Brey et al. manuscript submitted). This strategy obviates the need for enrichment of preexisting memory T cells, and hence is particularly attractive for therapy of HCMV infection after HSCT in the high-risk situation of an HCMV seropositive recipient and an HCMV seronegative donor. Our underlying concept thereby takes advantage of the fact that some HCMV-encoded proteins occur in high quantity on the surface of infected cells. However, in contrast to what has been achieved with comparable strategies for other viruses [14–19], in our work we learned that T cells redirected to HCMV-glycoprotein B (gB), despite strong activation, are not able to efficiently trigger apoptosis or lysis in cells infected with HCMV. Later we found that such strong protection of HCMV-infected cells against cytotoxic effector functions is at least partially mediated by HCMV-encoded anti-apoptotic proteins that are known to prevent suicide of infected host cells [20]. We hypothesized, however, that our approach could still inhibit HCMV replication independently from cytotoxic effector functions. This assumption is based on reports showing an important role for cytokines in the control of different viral diseases, including HCMV [21–25]. In the present study we confirm and extend this body of evidence by showing that CAR T cells can indeed efficiently inhibit HCMV replication in fibroblasts by secretion of interferon gamma (IFN-γ) and tumor necrosis factor (TNF).

Building on this observation, we investigated how we could improve our strategy of targeting HCMV by turning a potential weakness of our original gB-CAR design into an advantage. This gB-specific CAR, like many other CARs reported at that time, contained a CH2–CH3 Fc domain from IgG1. This domain, however, was shown to interact with Fc receptors (FcRs) and to trigger tonic signaling in T cells resulting in activation and inefficacy in vivo [26–30]. We hypothesized that in the case of HCMV, the Fc-mediated binding capacity of the CAR could represent an attractive opportunity to target this virus and to hamper possible immune escape due to antigen loss. Our hypothesis builds on the fact that HCMV blocks antibody-mediated immune responses by the expression of virally encoded Fc binding proteins on the surface of infected cells [31]. Importantly, the HCMV-encoded FcRs seem to essentially differ in their mode of binding to Fc domains compared to that of endogenous FcRs [32], which we speculated could enable us to target HCMV FcRs while sparing endogenous FcRs.

Our novel data now prove this hypothesis and, given the potent antiviral cytokines triggered by our CAR, we thus consider HCMV-FcR targeting an attractive additional means of combating HCMV.

## Methods

### Cell culture

Primary peripheral blood mononuclear cells (PBMCs) were isolated through leukapheresis of voluntary healthy donors by Ficoll density gradient centrifugation and stored in liquid nitrogen. Negative selection for isolation of CD3^pos^ T cells was performed using the Dynabeads Untouched Human™ T cell Kit (Life Technologies). T cells were further activated with Human T-Activator™ CD3/CD28 Dynabeads (Life Technologies) according to the manufacturer’s instructions (25 µl beads were used per 1 × 10^6^ T cells) and cultured in a medium consisting of RPMI GlutaMAX™ (Life Technologies) supplemented with 10% FCS (fetal calf serum, Sigma-Aldrich), 100 U/ml penicillin and 100 µg/ml streptomycin (both Life Technologies), i.e., a medium mix called “R-10”, plus 200 U/ml IL-2 (Peprotech). CD3/28-activated T cells were split every second day and cultured at densities between 0.5–1 × 10^6^ cells/ml. Primary human foreskin fibroblasts (HFF) were isolated from the foreskins of circumcised donors by mechanic disruption followed by enzymatic digestion with 5 mg/ml Collagenase D, 25 U/ml Dispase, and 0.05% Trypsin/EDTA. HFF were cultured in an R-10 medium and used between passage 10 and 18. Written informed consent was obtained from every donor’s parents, and the study protocols were approved by the ethics committee (Friedrich-Alexander-Universität Erlangen-Nürnberg no. 2247, Medizinische Universität Wien no. 514/2011). The hybridoma cell line “gB 27-287” [33] and the cell line IIA1.64 [34] were cultured in the R-10 medium. Neutralizing antibodies for IFN-γ (clone NIB42, Biolegend) and TNF (Infliximab; Centocor Ortho Biotech), and the respective isotype controls MOPC-21 and an anti-CD20 antibody (Rituximab, MabThera^®^; Roche) were added to HFF cultures as indicated.

### Construction of CARs

The gB-CAR was constructed using a single-chain variable fragment (scFv) derived from the antibody 27–287, as previously described [13]. For construction of CARs with different IgG-Fc parts, several mutations were introduced, as previously described [26, 35], into the spacers containing the Fc domain of either IgG1 or IgG4, i.e., substitution of ELLG (pos. 116–120) → PVA and S134A in IgG1-Fc (Uniprot ID P01857), and substitution of EFLB (pos. 113–116) → PVA in IgG4-Fc (Uniprot ID P01861). All CAR constructs were generated using the Gibson Assembly Kit (New England Biolabs) according to the manufacturer’s protocol. The resulting constructs were further amplified by PCR and used as templates for in vitro transcription.

### Viruses

Generation of infectious HCMV supernatants was performed by infecting semi-confluent HFF with the respective HCMV strain [multiplicity of infection (MOI) 0.1]. After 4 h, the remaining supernatant was discarded and fresh medium was added after washing it three times with R-10 to remove viral particles. Infected cells were cultured up to 14 days to achieve a high concentration of viral particles in the supernatant, which was subsequently harvested by centrifugation (2000 rpm, 10 min, 4 °C) and stored at − 80 °C until further use. Determination of the virus titer was performed by the limiting dilution method according to Reed and Munch. Infection of HFF for experiments with the respective HCMV strain was performed by 4-h co-incubation of the cells with thawed virus supernatant at an MOI 0.3 and 5 as indicated. Afterwards, the remaining supernatant was discarded and 3 washing steps with the R-10 medium were performed to remove remaining viral particles. Fresh medium was added and cells were further cultured at 37 °C. The HCMV strain AD169 encoding green fluorescent protein (GFP) was kindly provided by M. Marschall (Universitätsklinikum Erlangen, Germany), and the GFP-encoding HCMV strain Towne was a gift from B. Plachter (Universitätsmedizin, Johannes Gutenberg Universität Mainz, Germany).

### Flow cytometric analysis

To detect CARs on T cells and gB on HFF, the cells were first pre-incubated with 100 µg/ml murine IgG1**-**κ (clone MOPC21, Sigma-Aldrich) (10 min at 4 °C) before addition of the antibodies in order to prevent unspecific antibody-binding. In contrast, to detect FcRs on HFF and IIA1.64 cells, the cells were either not blocked before antibody addition or blocked, where indicated, with 10% human serum of an HCMV-negative individual (10 min, 4 °C). CAR expression in T cells was detected by incubation with a biotinylated anti-human IgG mab (clone JDC-10, Southern Biotec), followed by 2 washing steps and further incubation with PE-conjugated streptavidin (eBioscience). HCMV-gB was detected by incubation with supernatant from the hydridoma cell line gB-27-287 (final dilution 1:1) [33], followed by 2 washing steps and further incubation with a PE-conjugated anti-mouse antibody (eBiosicience). For detection of FcRs on the surface of HFF and IIA.64 cells, 0.5 × 10^5^ cells were incubated (without or with serum blocking, as indicated) with a fusion protein of IgG1-Fc coupled to CTLA4, termed CTLA4-IgG (5 µg/ml final concentration) (Bristol-Meyers Squibb Pharmaceuticals). After 2 washing steps, PE-conjugated anti-CTLA4 antibody (clone 14D3, eBioscience) was added to the cells. 7 AAD (eBioscience) was routinely added just before flow cytometric analysis in order to discriminate between viable and dead cells. All antibodies were incubated for 30 min at 4 °C. The washing buffer consisted of PBS (Life Technologies) supplemented with 10% FCS and 0.02% sodium azide (Merck KGaA). Flow cytometry was performed using a BD LSR Fortessa (BD Biosciences), and data were analyzed with FlowJo software (FlowJo Llc.). Cell counting was performed with Accucheck counting beads (Life Technologies), whereby dead cells were excluded by staining with propidium iodide.

### In vitro transcription and mRNA electroporation

The mRNAs encoding the CARs were generated by in vitro transcription from either 1 µg of linearized plasmid DNA or 50–200 ng of purified PCR products. In vitro transcription was performed using the mMessage mMachine T7 Ultra Kit (Ambion) according to the manufacturer’s protocol. In vitro transcribed mRNAs were purified with an adapted protocol using the RNeasy Kit (Qiagen). Briefly, RLT buffer from the kit and 1% beta-mercaptoethanol were added followed by the addition of absolute ethanol. After loading the sample onto an RNeasy column, purification was performed according to the manufacturer’s instructions. For electroporation of the mRNAs, 5 × 10^6^ cells were washed once with RPMI medium (Life Technologies) and once with Optimem (Life Technologies) (450×*g*, 5 min) to remove the remaining FCS and phenol-red. Afterwards, cells were resuspended in 100 µl Optimem, transferred into 4 mm electroporation cuvettes (VWR International GmbH) and electroporated with 10 µg mRNA using the Gene Pulser (Biorad) square wave protocol (500 V, 5 ms). The cells were used for in vitro assays 18–20 h after mRNA electroporation.

### ELISA

Supernatants were generated by co-culturing effector and target cells at an effector to target ratio of 2:1 in flat-bottom plates for 4 h at 37 °C. Prior to addition of the effector cells, the target cells (IIA1.64 or HFF) were routinely blocked with human serum of an HCMV-negative individual (10% final concentration, 15 min at 4 °C). Blocking with human serum was only omitted where indicated. The supernatants were collected, centrifuged (1600 rpm, 7 min) to remove remaining cells and stored at − 20 °C. IFN-γ in the supernatants was quantified using the Human IFN gamma ELISA Ready-SET-Go!^®^ Kit (eBioscience), and TNF was quantified using the Human TNF-α ELISA development kit (Mabtech). The limit of detection for IFN-γ and TNF was 4 and 13 pg/ml, respectively.

### Statistical analysis

Statistical significance was calculated using the paired two-tailed and the ratio paired two-tailed Student’s *t* test, as indicated in the figure legends (*** = p < 0.001; ** = p < 0.01; * = p < 0.05; ns = p > 0.05).

## Results

### gB-CAR T cells can inhibit HCMV replication independently from cytotoxicity

We previously generated a gB-specific CAR and showed that this CAR triggers T cell activation in response to HCMV infected cells. Since this does not result in substantial lysis of the infected cells, we asked if the CAR T cells could still efficiently inhibit HCMV replication by secretion of cytokines.

**Fig. 1:**
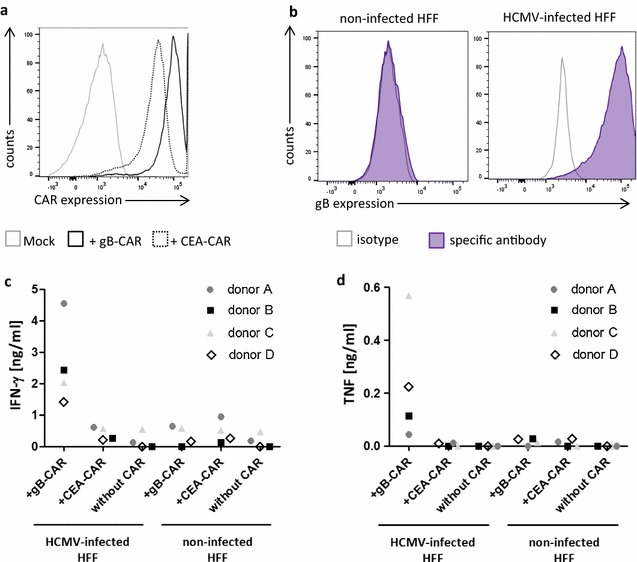
gB-specific CAR T cells secrete IFN-γ and TNF in response to HCMV-infected cells. **a** Flow cytometric analysis of CAR expression in anti-CD3/CD28-activated T cells 1 day after mRNA electroporation. CARs were detected via an anti-human-IgG antibody. **b** Flow cytometric analysis of HCMV-gB expression in uninfected HFF and in HFF 4 days after infection (AD169; MOI 5). **c** and **d** Absolute amounts of IFN-γ (**c**) and TNF (**d**) secreted by anti-CD3/CD28-expanded T cells 1 day after electroporation of CAR-mRNA. T cells were co-cultured for 4 h with uninfected and infected HFF (day 4 p.i., AD169, MOI 5)

**Fig. 2:**
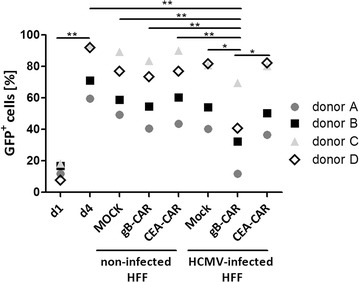
Supernatants of gB-specific CAR T cells inhibit HCMV replication in HFF. T cells were transfected with CAR-mRNA and 1 day later were co-cultured for 4 h with uninfected HFF or HFF 4 days after infection (AD169; MOI 5). The supernatants of these co-cultures (analysis shown in Fig. 1c) were added to fresh HFF concomitantly with the HCMV (AD169; MOI 0.3). 1 or 4 days later the HFF were trypsinized and harvested for flow cytometric determination of the fraction of GFP^pos^ HFF (n = 4; two-tailed Student’s *t* test)

As a first step, we harvested supernatants of co-cultures of infected and non-infected HFF with T cells expressing either a gB-specific CAR or a CAR with irrelevant specificity [carcinoembryonic antigen (CEA)-specific CAR]. CAR expression in the T cells is depicted in Fig. 1a, and HCMV-gB expression in HFF is shown in Fig. 1b. Figure 1c and d illustrate that only CAR T cells expressing the gB-CAR specifically respond to HCMV-infected HFF and secrete IFN-γ and lower amounts of TNF. The blocking capacity of these supernatants was then tested in a subsequent experiment, in which HFF were infected with recombinant HCMV (strain AD169) encoding GFP under an immediate early promoter. This allowed for quantification of the fraction of infected HFF (green cells) by flow cytometry starting from 1 day after infection. Infection dose was low (MOI 0.3) in order to warrant that only a small fraction of HFF was initially infected (7.9–18.2% GFP^pos^ HFF on day 1; Fig. 2). Until day 4 after infection almost all of the HFF became GFP^pos^ (59.5–93.7%) due to reinfection with the newly replicated virus starting from day 3 after infection. This viral spread until day 4 was significantly inhibited (11.8–69.5% GFP^pos^ HFF) if cell-free supernatants from gB-CAR T cells (donors A–D) co-cultured with infected HFF were added simultaneously with the viral supernatant. Supernatants from the control conditions (T cells minus/plus irrelevant CAR, or co-culture with non-infected HFF) had no significant effect (Fig. 2). Additional file 1: Figure S1A and B depict the kinetics of infectious virus production in HFF (cell associated versus released particles after infection with HCMV at two different doses). This experiment was the basis for designing the above blocking trial and showed that new infectious virus particles first appear on day 3. The majority of the virus present on day 3 is cell associated. Release of free virus particles is low on day 3, but increases 100–1000-fold until day 5, whereas cell-associated particles increase only slightly.

### Inhibition of HCMV replication by CAR T cells is mediated mainly by IFN-γ and TNF

**Fig. 3:**
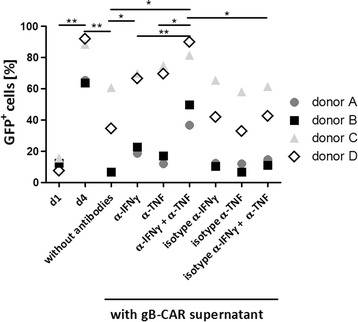
Blockade of IFN-γ and TNF neutralizes the inhibitory effect of CAR T cells. The supernatants of the gB-specific CAR T cells co-cultured with infected HFF (which were already used for the experiments shown in Figs. 1c and 2) were added to fresh HFF concomitantly with HCMV (AD169; MOI 0.3) and different blocking antibodies or isotype antibodies as indicated (30 µg/ml each). The same antibodies were added again on day 2 after infection. The percentages of infected HFF (GFP^pos^) were determined by flow cytometric analysis on day 4 (n = 4; two-tailed Student’s *t* test)

In the next step we wanted to investigate which factors secreted by the gB-CAR T cells inhibit the replication of HCMV. Early studies showed that type I interferons but also the T cell secreted cytokines IFN-γ and TNF can have a strong antiviral capacity [24, 25]. So, we repeated the infection experiment explained above with supernatants from gB-CAR T cells plus/minus neutralizing antibodies against IFN-γ and TNF. Figure 3 illustrates that the inhibitory effect of the co-culture supernatants (T cell donors A-D) on HCMV replication in HFF until day 4 was almost completely abrogated by the combined neutralization of IFN-γ and TNF. The neutralization of either IFN-γ or TNF alone had a smaller effect, and, as expected, the addition of the isotype control antibodies had no effect on the inhibition of HCMV replication by the supernatants.

Overall these data demonstrate that the gB-CAR mediates inhibition of HCMV replication independent of cytotoxic effector functions via the combined action of IFN-γ and TNF, which are released by the activated CAR T cells.

### Fc spacer domains in CARs enable additional targeting of HCMV-encoded FcRs

Our original gB-specific CAR construct contained a CH2–CH3 Fc domain that was meanwhile recognized to abrogate CAR T cell function in vivo [26–30]. However, since HCMV encodes at least four different IgG-Fc binding proteins in order to escape from antibody-mediated immune responses [31], we speculated that this CH2–CH3 domain after mutation could be particularly attractive for targeting HCMV.

**Fig. 4:**
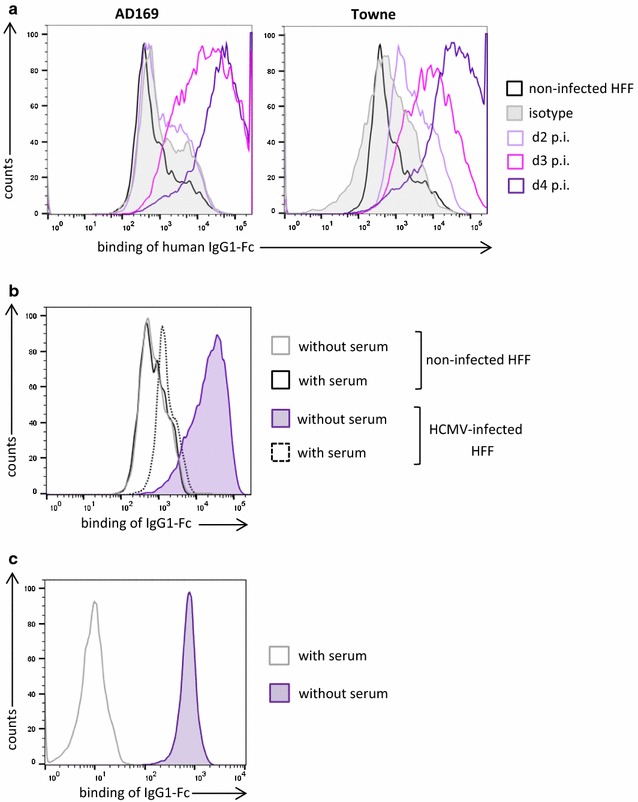
Expression of HCMV-FcRs in infected HFF. **a** Flow cytometric analysis of the capacity of HFF for binding of IgG1-Fc (i.e., CTLA4-IgG) at different days after infection (MOI 5). Shown is one representative experiment (n = 3). **b** Binding of CTLA4-IgG to non-infected and infected HFF (AD169; MOI 5; day 4 p.i.) and **c** IIA1.64 cells with and without prior addition of 10% human serum. Shown is the flow cytometric analysis of one representative experiment (n = 3)

**Fig. 5:**
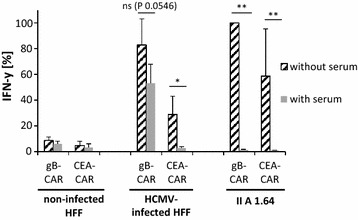
HCMV-FcR expressed in infected HFF can trigger CAR T cells. IFN-γ secretion from gB- and CEA-specific CAR T cells after co-culture with non-infected HFF or HFF 4 days after infection (AD169; MOI 5) or a IIA1.64 cells. CH2–CH3 dependence of CAR T cell activation was analyzed by the addition of 10% human serum as indicated. IFN-γ levels were normalized for each donor to the value obtained with the co-culture of gB-specific CAR T cells with IIA1.64 cells (mean ± standard deviation; n = 3, three different donors; ratio paired two-tailed Student’s *t* test)

As a first step we analyzed the kinetics of HCMV-FcR expression by determining the capacity of infected HFF for binding of human IgG1-Fc. Figure 4a illustrates that IgG1-Fc could strongly bind to HFF starting from day 3 after infection with HCMV strains AD169 and Towne. For these experiments we used IgG1-Fc fused to CTLA4, which was detected by second-step staining with an anti-CTLA4 antibody. Binding of the CTLA4-Fc to infected HFF was completely blocked by pre-incubation of the cells with human serum, demonstrating that the interaction is mediated by the Fc domain (Fig. 4b). The same procedure plus/minus prior serum blocking was applied for detection of human CD64 in the murine cell line IIA1.64 [34] which expressed high levels of CD64 (Fig. 4c) and was used for control. In a next step we co-cultured CAR T cells with infected or non-infected HFF or IIA1.64 cells and determined the secretion of IFN-γ (Fig. 5). IFN-γ levels were normalized to the levels obtained with the CD64^high^ control cell line IIA1.64. A CEA-specific CAR was included as a non-relevant CAR that could only be activated by the infected HFF via the integrated CH2–CH3 spacer. Figure 5 shows that both the gB-specific and the CEA-specific CAR, in the absence of serum blocking, efficiently recognized the CD64^high^ IIA1.64 cells and the infected HFF, whereas there was no specific response towards uninfected HFF. As expected, pre-incubation of the target cells with human serum completely blocked IFN-γ production in T cells, when the interaction with target cells was only mediated via the CH2–CH3 domain in the CAR. This was the case when the CAR T cells were co-cultured with IIA1.64 cells or when CEA-specific CAR T cells were co-cultured with infected HFF. However, when the T cells were directed against infected HFF via the gB-specific CAR, which could additionally interact via the gB-specific scFv, then IFN-γ secretion was only partially blocked by the human serum. Notably, the same T cells were completely blocked by human serum in co-culture with IIA1.64 cells.

Our data confirm two points: First, that HCMV-encoded FcRs similarly to gB occur on the surface of infected cells starting from day 3 after infection and hence could serve as target antigens. Second, the data show that the HCMV-FcRs indeed interact with and efficiently trigger activation in T cells expressing CARs with integrated CH2–CH3 domains.

### HCMV-encoded FcRs can be targeted separately from endogenous FcRs

Since the recognition of human endogenous FcRs by the CH2–CH3 domain results in reduced efficacy of the CAR T cells in vivo, a wild-type CH2–CH3 domain for recognition of HCMV-FcRs is not an option. However, there is evidence that the mode of interaction of HCMV-FcRs could substantially differ from that of endogenous FcRs [32]. We hypothesized that introducing specific mutations inhibiting the interaction of the CH2–CH3 domain with endogenous FcRs (except the neonatal FcR) could still allow for interaction with HCMV-FcRs.

**Fig. 6:**
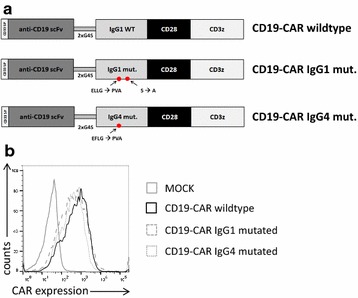
Expression of CARs with mutated CH2–CH3 domains. **a** Schematics of the CARs with different CH2–CH3 variants. An FMC63-based scFv with a signal peptide from CD33 was fused via a linker containing the amino acids GGGGSGGGGS (2 × G4S) and different CH2–CH3-spacer domains to a CD28/C3zeta-based CAR signaling backbone. The mutations are indicated (positions according to Uniprot IDs P01857 and P01861 of human IgG1 and IgG4, respectively). **b** Expression of the different CARs in anti-CD3/CD28-expanded T cells 1 day after electroporation of the respective mRNAs. Expression was detected via an anti-human-IgG antibody and analyzed by flow cytometry

**Fig. 7:**
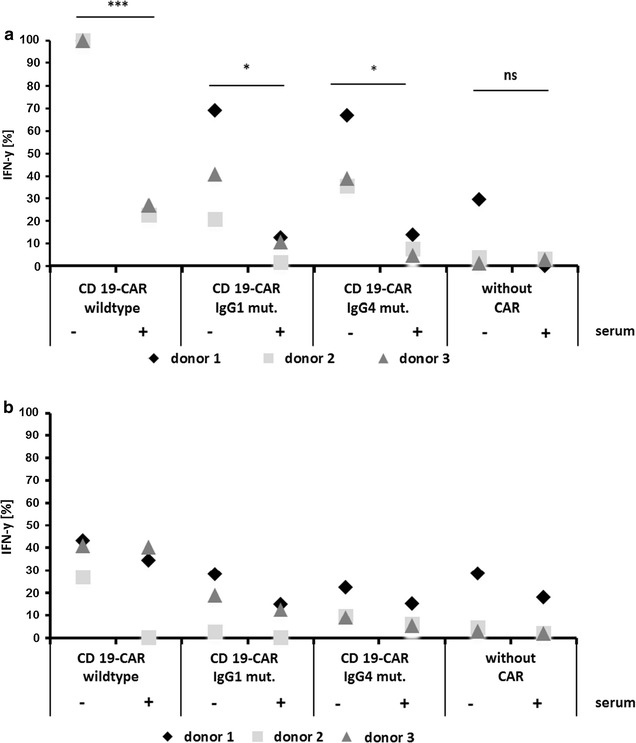
HCMV-FcRs can be specifically recognized by mutated CH2–CH3 domains. Secretion of IFN-γ from different CAR T cells co-cultured for 4 h with **a** HCMV-infected HFF (AD169; MOI 5; 4 days p.i.) and **b** non-infected HFF. CH2–CH3 dependence of CAR T cell activation was analyzed by pre-incubation of the HFF with 10% human serum (final concentration) as indicated. IFN-γ levels were normalized for each donor to the value obtained in absence of human serum in the co-culture of T cells expressing the CAR with the wild-type CH2–CH3 domain (1.0–3.2 ng/ml) (n = 3, three different donors; ratio paired two-tailed Student’s *t* test)

Consequently, we generated CARs with mutated CH2–CH3 domains of IgG1 and IgG4, which were previously reported to not interact with endogenous FcRs and to rescue CAR function in vivo [26, 35]. These CH2–CH3 domains (Fig. 6a) were integrated into a non-gB CAR, which was directed against CD19, and not CEA, since it turned out that mutated CH2–CH3 domains further reduced the expression of the CEA-specific CAR, which already showed reduced expression on its own. This unwanted reduction was not observed with the CD19-specific CAR (Fig. 6b). The CD19-specific CARs with either the mutated Fc domain of IgG1 or of IgG4 indeed mediated the recognition of HCMV-infected HFF, which again was inhibited by pre-incubation of the HFF with human serum (Fig. 7a). The cytokine levels achieved with CARs containing the mutated Fc domains, however, were reduced compared to the CAR with a wild-type CH2–CH3 (Fig. 7a). Notably, also the antigen independent background secretion of IFN-γ in the co-cultures with non-infected HFF was reduced in T cells with mutated CH2–CH3 domains (Fig. 7b), possibly indicating a generally reduced capacity for cytokine secretion in the cells resulting from expression of an unstable mutated protein domain.

These experiments prove that CARs with mutated IgG1- and IgG4-Fc domains indeed allow for recognition of HCMV-infected cells without targeting endogenous FcRs.

## Discussion

The concept of an HLA-independent immunotherapy is under investigation for HIV and hepatitis viruses [14–19]. The strengths of such an approach lie in the circumvention of expanding memory T cells and in the broad applicability independent of HLA types. We thus previously generated a CAR directed against HCMV-gB, which can efficiently redirect T cells towards HCMV-infected cells [13]. A characteristic of HCMV, however, is the strong protection of the infected host cells against cytotoxic effector functions, as shown in our previous study [20]. In that study we found that T cell degranulation does not result in lysis of the infected cells, and this could be attributed, at least partially, to direct blockade of cell death induction by viral anti-apoptotic proteins UL37 × 1 and UL36.

It has long been known that non-cytolytic effector functions play an important role in viral defense, whereby the interferon response makes a major contribution [21, 36]. This type of innate immune defense has come into focus again only recently as an important mechanism against hepatitis viruses and has also been extensively investigated for HCMV [22, 37, 38]. Notwithstanding the role of type I interferons, the strong antiviral effect of IFN-γ was demonstrated early on for HCMV in vivo, and a polymorphism in the IFN-γ gene was correlated with susceptibility to HCMV infection in kidney transplant recipients [24, 39, 40]. In fact, protection is mediated not only by interferons alone, but by the combination with other cytokines [41]. For example, TNF cooperates with IFN-α in inducing interferon-stimulated gene expression and additionally mediates interferon-independent antiviral activity against human hepatitis viruses [37, 42]. For murine CMV synergies between TNF and IFN-γ have been previously demonstrated [25, 43]. Our data with human CAR T cells now confirm and extend these prior observations. We show that these cells efficiently recognize infected cells and produce IFN-γ and smaller amounts of TNF, when directed to viral proteins on the surface of infected cells. Again, it is the combination of IFN-γ and TNF that makes the major contribution to efficient inhibition of HCMV replication in HFF, as we show in our experiments with cytokine neutralizing antibodies.

In our previous study, we found that gB occurs on the surface of infected cells during the replication cycle only when the cells are already strongly protected against cytolytic effector functions by the viral anti-suicide machinery. We now show that gB-specific CAR T cells can nevertheless inhibit HCMV replication by the secretion of high levels of IFN-γ and TNF. Given these results, we have also furthered our approach in a separate study with a bispecific antibody construct based on the BiTE^®^ platform (Brey C. et al., manuscript submitted), and have included HCMV-encoded FcRs as additional targets.

We hypothesized that our original gB-CAR, which contained a CH2–CH3 domain of IgG1, could function like other bispecific CARs that have OR-gate function [44–46]. This OR-gate function means that the CAR could not only recognize HCMV-gB via its scFv domain, but also the HCMV-FcRs via its CH2–CH3 domain, which could possibly increase function and/or hamper immune escape by antigen loss. HCMV encodes at least four different Fc-binding proteins that block IgG-mediated immune responses by binding to the Fc portions of antibodies [31]. All primary HCMV isolates encode for Fc-binding proteins, hinting at a strong selective pressure, and we speculate that their capacity for binding to CH2–CH3 domains should remain conserved due to their particular biological role. HCMV-FcRs can bind different immunoglobulin classes, including rabbit IgG in the case of gp34 and gp68, and they are expressed at different levels on the surface of infected cells [31]. Figure 4a shows that HFF can strongly bind IgG1-Fc 3 days after infection with AD169. The same is true if the CH2–CH3 domain is incorporated into the CAR backbone, and indeed this domain mediates recognition of HCMV-infected cells and confers OR-gate function (Fig. 5).

Notably, we could inhibit this CH2–CH3 mediated interaction by pre-incubation of the target cells with human serum. Human serum is frequently used for blockade of FcRs and the mechanism of action seems obvious. However, what is frequently forgotten is the fact that FcR blockade is mediated by multivalent aggregates of immune complexes that have high affinity and are formed in serum upon freezing or heat inactivation [47, 48]. Such aggregates do not exist in vivo, except in some pathological conditions, and hence this inhibition is artificial. In the body, high concentrations of monomeric IgG in serum might actually saturate the high-affinity FcR CD64 (K_D_ 10^−10^–10^−8^ M), whereas CD32 and CD16 (K_D_ 10^−7^–10^−5^ M) can only efficiently bind aggregated or membrane bound IgG [49, 50]. Still, CD64 is not blocked by highly concentrated serum IgG, but can specifically respond to IgG that is bound to target cells. The same is true for HCMV-FcRs (K_D_ ~ 10^−7^ M for gp68 [32]) which are not blocked by the high IgG concentration in serum, but can specifically catch and endocytose HCMV-specific IgG that has bound to the infected cells [51]. Importantly, although this mechanism of HCMV-FcRs protects infected cells from antibody- and complement-dependent elimination, our data show that it does not prevent the recognition by CAR T cells.

Just as CARs with incorporated Fc domain can recognize HCMV-FcRs, they can also bind to endogenous FcRs. Such interaction with endogenous FcRs, however, is known to trigger tonic activation and activation-induced cell death and thereby to cause inefficacy of the CAR T cells in vivo [26–30]. Further, we speculate that auto-stimulation and/or fratricide due to expression of the low-affinity FcR CD16 on a fraction of activated T cells themselves might be part of the problem [52–54]. Incorporation of the wild-type CH2–CH3 domain for additional targeting of HCMV-FcRs hence was not an option. However, since there is evidence that HCMV-FcRs might interact with IgG in different ways from endogenous FcRs [32], we hypothesized that HCMV-FcRs could be specifically targeted without recognition of endogenous FcRs. Mutations were previously introduced into specific sites of the CH2–CH3 domain of IgG1 and IgG4 to prevent the interaction with endogenous FcRs and thereby restore the CAR T cell function [26–30]. We integrated these reported CH2–CH3 domains into CARs and tested if the T cells could still recognize HCMV-infected cells. As Fig. 7a illustrates, this is in fact the case.

Notably, the capacity for cytokine production seemed reduced in T cells expressing CARs with the mutated CH2–CH3 domains. We assume that this is not a result of reduced affinity, which would lower the sensitivity, but rather an unspecific effect of the mutations on protein folding and stability. Our assumption is based on the observation that the antigen-independent background of cytokine secretion also tended to be lower in the T cells with mutated CH2–CH3 compared to wild-type CH2–CH3 (Fig. 7b), and that we could hardly express CARs encompassing mutated CH2–CH3 and the CEA-specific scFv. Notably, the CEA-specific scFv by itself seemed to destabilize the protein and/or impede protein folding, since CEA-CAR expression levels were generally lower compared to the gB-specific CAR (Fig. 1a). The same mutated CH2–CH3 domains, however, could be expressed in the context of the FMC63-based CD19-specific scFv. Still, this domain constellation could also possibly trigger an unfolded protein response pathway in the T cells, which could reduce protein expression, as we have experienced with several other unstable proteins (unpublished observations). We believe that in further development, beyond increasing affinity and optimizing the CAR design, the introduction of stabilizing mutations into CH2–CH3 domains is needed to fully exploit the potential of HCMV-FcR targeting.

## Conclusions

Overall, our study shows that the FcRs encoded by HCMV are an attractive novel target for HLA-independent immunotherapeutic approaches. We demonstrate that, beyond the native biological function of HCMV-FcRs of blocking antibody-mediated immune responses, these proteins may represent a viral Achilles heel allowing for efficient retargeting of T cells and their cytokine-mediated antiviral effects toward HCMV-infected cells. Instead of engineering novel antibodies we employ mutated endogenous IgG-Fc domains to target HCMV-FcRs. This strategy is associated with only minimal potential immunogenicity, since very few mutations are required for disruption of the IgG-Fc interaction with endogenous FcRs. Our study also indicates that the Fc spacer in CARs and possibly also bispecific antibody constructs can have a new and important role in the context of HCMV antiviral therapy.

## Additional file

**Additional file 1: Figure S1.** Replication kinetics of HCMV in HFF. Shown is the number of infectious virus particles obtained from 1x10^6^ HFF at different time points after infection with HCMV (AD169; MOI as indicated). **(A)** Infectious particles released into the supernatant. **(B)** Cell associated infectious particles obtained from centrifuged supernatant after ultrasonic homogenization of the HFF.

## Abbreviations

CAR: chimeric antigen receptor
CEA: carcinoembryonic antigen
FcR: Fc receptor
gB: glycoprotein B
GFP: green fluorescent protein
HCMV: human cytomegalovirus
HFF: human foreskin fibroblasts
HSCT: hematopoietic stem cell transplantation
MOI: multiplicity of infection
pfu: plaque-forming units
scFv: single-chain variable fragment
SOT: solid organ transplantation
