# Supplementary material for: Turning the tables on cytomegalovirus: targeting viral Fc receptors by CARs containing mutated CH2–CH3 IgG spacer domains
Source: J Transl Med. 2018 Feb 8;16:26. doi: 10.1186/s12967-018-1394-x (PMC5804023; doi:10.1186/s12967-018-1394-x)
Supplement: Supplementary file 1 — Additional file 1: Figure S1. Replication kinetics of HCMV in HFF. Shown is the number of infectious virus particles obtained from 1x10^6^ HFF at different time points after infection with HCMV (AD169; MOI as indicated). (A) Infectious particles released into the supernatant. (B) Cell associated infectious particles obtained from centrifuged supernatant after ultrasonic homogenization of the HFF. [file 12967_2018_1394_MOESM1_ESM.pdf]

Additional Figure S1

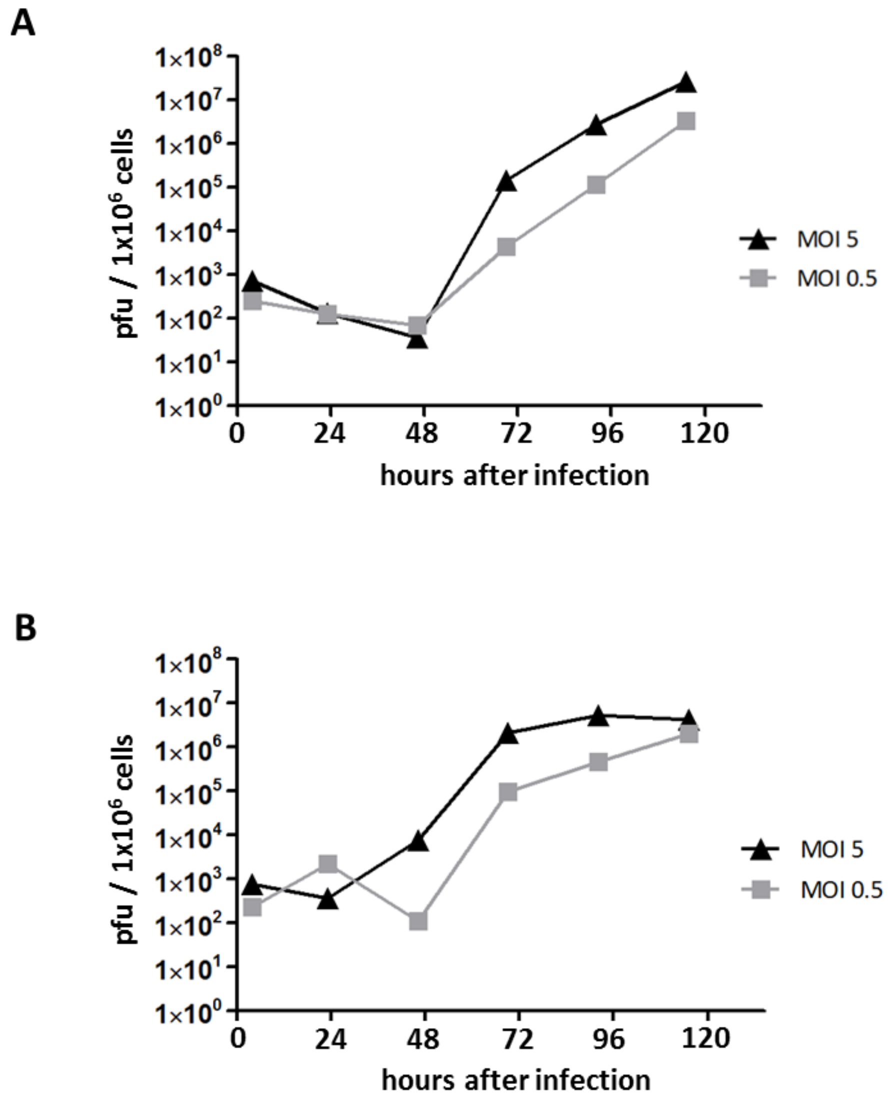

Replication kinetics of HCMV in HFF. Shown is the number of infectious virus particles obtained from $1x10^6$ HFF at different time points after infection with HCMV (AD169; MOI as indicated). **(A)** Infectious particles released into the supernatant. **(B)** Cell associated infectious particles obtained from centrifuged supernatant after ultrasonic homogenization of the HFF.
